# Microstructural Effects of Melt Electrowritten-Reinforced Hydrogel Scaffolds for Engineering Thick Skin Substitutes

**DOI:** 10.1021/acsabm.4c01541

**Published:** 2025-03-25

**Authors:** Ferdows Afghah, Mine Altunbek, Mahdiyeh Zahrabi, Bahattin Koc

**Affiliations:** † Sabanci University Nanotechnology Research and Application Center, Istanbul 34956, Turkey; ‡ Sabanci University Faculty of Engineering and Natural Sciences, Istanbul 34956, Turkey; § Sabanci University Integrated Manufacturing Technologies Research and Application Center, Istanbul 34906, Turkey

**Keywords:** melt electrowriting, microstructure, scaffold design, cell alignment, vascularization, mechanical properties, skin tissue engineering

## Abstract

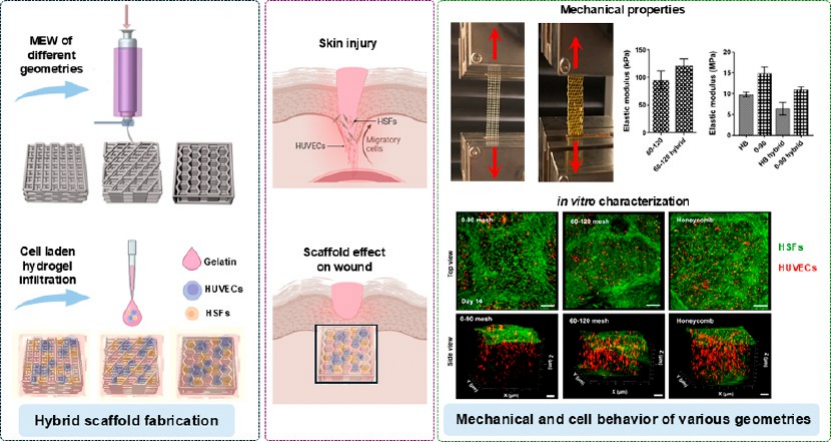

Engineering thick skin tissue substitutes resembling the physiochemical and mechanical properties of native tissue is a significant challenge. Melt electrowriting (MEW) is a powerful technique with the capability of fabricating highly ordered structures with fine fiber diameters, closely replicating the native extracellular matrix (ECM). In this study, we constructed melt electrowritten porous polycaprolactone (PCL) scaffolds with three different geometries by depositing fibers at 0–90 and 60–120° in a mesh structure and in a honeycomb-like orientation to assess the effects of the microstructure on the mechanical strength of the scaffold and cellular behavior. These scaffolds were subsequently infilled with gelatin hydrogel, encapsulating human skin dermal fibroblasts (HSFs) and human umbilical vein endothelial cells (HUVECs). Mechanical tensile tests revealed that the honeycomb microstructure of the hybrid PCL/gelatin scaffold exhibited greater elongation at failure, along with an acceptable elastic modulus suitable for skin tissue applications. All scaffolds provided a cytocompatible microenvironment that maintained over 90% cell viability and preserved typical cell morphology. HSFs were guided through the PCL fibers to the apical surface, while HUVECs were distributed within the gelatin hydrogel within the hybrid structure. Additionally, HSFs’ alignment was regulated by the scaffold geometry. Notably, the expression of CD31 in HUVECs—a key transmembrane protein for capillary formation—increased significantly over a 14 day incubation period. Among those, 0–90° mesh and honeycomb geometries showed the greatest effects on the upregulation of CD31. These findings demonstrate that the microstructural guidance of HSFs and their interaction with HUVECs in hybrid structures play a crucial role in promoting vascularization. In conclusion, the honeycomb MEW-gelatin hybrid scaffold demonstrates significant potential for effectively replicating both the mechanical and physicochemical properties essential for full-thickness skin tissue substitutes.

## 1 Introduction

Demands for skin tissue substitutes have increased significantly over the last few decades due to the vast majority of large and deep skin loss caused by burns, trauma, and diabetic skin ulcers.^1^ Initial solutions focused on skin substitutes, ranging from xenografts to allografts. These were later followed by advancements in regenerative medicine, leading to the fabrication of skin equivalents from natural and synthetic materials to address the limitations of the former traditional treatments.^2^ A promising tissue scaffold must possess a stiff and fibrous microstructure mimicking extracellular matrix (ECM),^3^ a hydrophilic surface for cell spreading, elongation, and proliferation and interconnected pores allowing the transportation of nutrients and oxygen.^4^ Additive manufacturing technology, commonly known as three-dimensional (3D) printing, has come into the spotlight owing to its ability to fabricate 3D constructs that mimic native structures at relatively low cost, with high precision and speed. This technology enables the fabrication of intricate geometrical structures.^5,6^ Although 3D printing can produce scaffolds with highly ordered and controlled architectures, the fiber diameters are often too large, resulting in slow degradation rates and delayed replacement by cell-produced ECM. Melt electrospinning writing (MEW) is a promising 3D fabrication method that produces micrometer-scale fibers by combining the features of fused deposition modeling (FDM), a form of melted thermoplastic material extrusion printing, with solution electrospinning^7^. This approach leverages computer-aided design (CAD) modeling of FDM, enabling precise control over fiber deposition, geometry, and pore size. Meanwhile, implemented high voltage results in the formation of micron-sized fibers.^8,9^ Additionally, the final structure contains interconnected pores that promote nutrient and oxygen transfer, as well as cell migration. Fabricating scaffolds with filament diameters in the tens of microns mimics the ECM of the skin, providing a substrate that would guide cells while maintaining flexibility to facilitate integration with surrounding tissues.^10,11^ Various thermoplastic polymers have been utilized in the MEW of scaffolds for different tissue engineering applications.^12^ Polycaprolactone (PCL) is one of the most widely used polymers because of its biocompatibility, low melting temperature, availability, and favorable mechanical properties.^13^ It has already been employed as a skin scaffold, successfully supporting fibroblast cell infiltration.^10,14^ A study by Chong et al.^15^ demonstrated that MEW of PCL can produce structures with high strength and enhanced elasticity, making it well-suited for skin tissue engineering. In another study by Dalton’s group, a bilayer scaffold combining MEW with a solution electrospun membrane was demonstrated to create a properly differentiated full thickness skin model with distinct dermis and epidermis layers, exhibiting mechanical properties closely resembling those of native human skin.^16^ Dubey et al.^17^ investigated the use of MEW PCL meshes to enhance cell-laden hydrogels for skin tissue engineering, employing a methodology similar to that used in this study. Their research focused on delivering periostin to promote wound healing and developing mechanically robust constructs.

Vascularization is one of the most critical and challenging aspects of thick 3D printed tissue constructs. Without proper vascularization, the flow of oxygen, nutrients, and waste is inhibited. This limitation hinders the effective integration of thick tissue substitutes into host tissue, long-term survival, and function.^18−21^ To address this, various strategies have recently been explored for inducing vascularization in skin tissue engineering. A review by Iqbal et al.^22^ categorizes various approaches for enhancing vascularization in skin tissue engineered substitutes. For instance, Liu et al.^21^ demonstrated that incorporating phosphosilicate calcium bioglass can promote vascularization in full-thickness skin substitutes. Similarly, Limido et al.^23^ introduced a novel approach utilizing nanofat, which includes growth factors, ECM, and adipose tissue-derived stem cells showing promising results in promoting vascularization of full-thickness murine wounds. Interestingly, studies have shown that the density and stiffness of structures also influence vascularization.^24^ A common approach to promote or enhance vascularization in the scaffolds is the incorporation of endothelial cells.^25−28^ For example, Pourchet et al.^29^ developed a vascularized full-thickness bilayered skin construct of 5 mm height, using human umbilical vein endothelial cells (HUVECs).

Various hydrogels including collagen, blood plasma, alginate, gelatin, gelatin methacryloyl (GelMA), poly(ethylene) glycol (PEG)-based hydrogels, and fibrin have been used to encapsulate these cells to create perfusable and vascularized skin substitute models.^21,30,31^ Among these hydrogels, gelatin has gained increasing attention due to its biocompatibility, biodegradability, and the presence of cell adhesive arginine-glycine-aspartic acid (RGD) amino acid sequences, which enhance granulation and epithelialization by facilitating cell adhesion.^32^ The prolonged stability of gelatin is crucial for providing the necessary mechanical and structural support for cells during regeneration. Various cross-linking mechanisms are utilized to enhance the stability but the photopolymerization shows particular promise as it allows for the modulation of degradability and stiffness of the hydrogel through controlling the cross-linking.^33,34^ Visible light cross-linking offers great advantages with higher penetration depth compared to UV radiation, making it ideal for cross-linking thick hydrogel scaffolds.^35,36^ In this photopolymerization process, a ruthenium (Ru) complex and sodium persulfate (SPS) are commonly utilized as photocatalysts and photoinitiators, respectively.^37^ The cross-linking of gelatin hydrogels under visible light can facilitate a cytocompatible photo-cross-linking method with the potential applications in biofabrication strategies.

The microstructural design of scaffolds is another key factor in modifying their mechanical properties and guiding biological responses, such as cell alignment, and signaling, cell fate, and functionality.^38−43^ Most current research focuses on the effects of micropatterns in bone and cartilage tissue engineering applications.^44−46^ Nikkhah et al.^47^ demonstrated that GelMA micropatterns with varying geometrical dimensions could induce vascularization. They fabricated multiple parallel rectangular micropatterned constructs at various thicknesses, infiltrating endothelial cells within the GelMA hydrogel. The rectangular shape of the hydrogel guided the cell proliferation and alignment, ultimately leading to the formation of cord-like structures, a crucial early step in vascularization. Similarly, Zhu et el.^48^ reported a micropatterned electrospun fibrous scaffold that showed potential for blood vessel formation. Their study compared aligned and nonaligned fiber structures and demonstrated that aligned fibers facilitated the phenotype formation of HUVECs. In a more recent study, Yao et al.^41^ explored the effect of structural architecture on endothelial cell morphogenesis. By comparing random and honeycomb electrospun structures for culturing HUVECs, they found that the honeycomb geometry guided the cells to form a tubular structure, which is a precursor to vascularization. This underscores the critical role of the ordered microstructures in guiding cell arrangement and fate, a phenomenon that could be harnessed to manipulate cellular behavior for desired applications.

Compared to previous studies, in this study, the microstructural effects of three distinct MEW PCL mesh geometries, including 0–90 mesh, 60–120° mesh, and honeycomb structures in a hybrid design, were investigated for the construction of a mechanically robust, thick, and vascularized skin substitute. Human skin fibroblasts (HSFs) and HUVECs were cocultured within a visible light cross-linked gelatin hydrogel in the PCL/gelatin hybrid structure. The mechanical properties of the scaffolds, both with and without the hydrogel were assessed, and the effect on cell viability, and alignment, and vascularization were analyzed. Among the designs, the honeycomb structure exhibited higher elastic work and elongation at the break, making it particularly relevant for skin tissue engineering. Additionally, both the honeycomb and 0–90° mesh architecture demonstrated a significant effect on promoting the vascularization.

## 2 Materials and Methods

### 2.1 Materials

PCL CAPA 6400 (molecular weight of 37,000 g/mol) was acquired from Perstorp Ltd., UK. Sodium hydroxide (NaOH), gelatin type B, tris (2,2-bipyridyl) dichlororuthenium(II) hexahydrate (Ru), SPS, and Fetal bovine serum (FBS) were purchased from Sigma-Aldrich. HUVECs, HSFs, Dulbecco’s modified Eagle’s medium (DMEM), and vascular cell basal medium were bought from ATCC. Pen-Strep (10,000 Units/ml penicillin, 10,000 μg/mL streptomycin) and Trypsin–EDTA were obtained from Gibco, UK. Phosphate buffered saline (PBS) tablets were purchased from MP Biomedical, France.

### 2.2 CAD Modeling and Scaffold Fabrication via MEW

**Figure 1:**
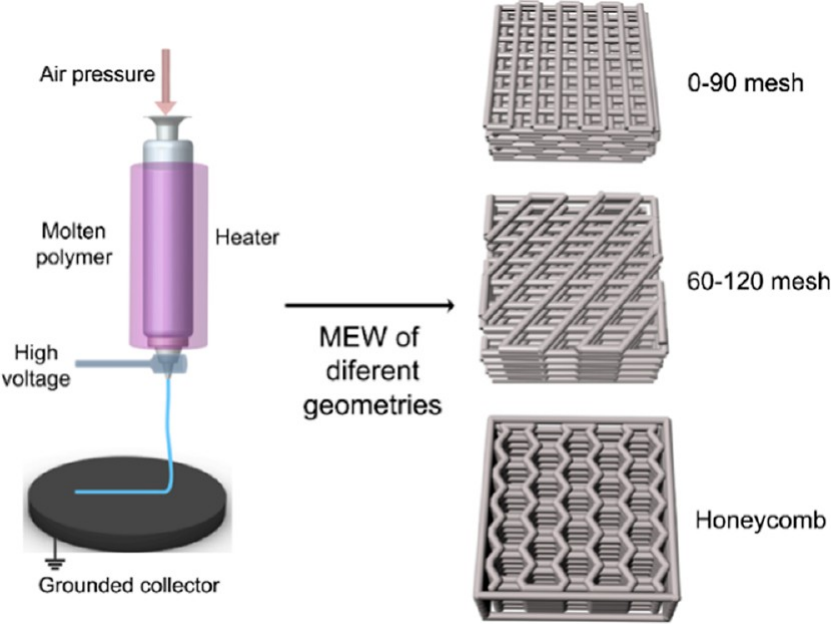
(A) Schematic representation for the melt electrowriting process. Air pressure is applied to the molten PCL, and with the aid of CAD design, micron-size filaments formed under high voltage are deposited onto a grounded collector in 0–90° mesh, 60–120° mesh, and honeycomb geometries.

Algorithms for continuous printing paths were developed for three different structures: meshes with 0–90 and 60–120° orientations and a honeycomb shape, denoted as HB. The 0–90° mesh was selected as one of the most commonly used geometries in tissue engineering. To investigate the effect of different fiber orientations, we also chose a 60–120° mesh. Additionally, we incorporated a honeycomb-like structure, characterized by its unique architecture with multiple angles and symmetrical orientation, as the third geometry. These geometries were expected to influence both the cell guidance and mechanical properties. These geometries were anticipated to influence both cell guidance and mechanical properties. The algorithms where then converted to G-codes using a Python script, which were subsequently loaded into MACH3 motion controller software (Newfangled Solutions). The melt electrowriting setup was a custom-made platform developed on a 3-axis printer, as previously explained.^7^ The setup included two heating units controlled by an Arduino Mega microcontroller, a 10 mL syringe with a 250 μm diameter nozzle mounted on the print head, an air dispensing system (Musashi Engineering Inc., Japan), and a high voltage generator (Gamma High Voltage Research, FL). The collector was grounded, and the nozzle was connected to a high-voltage generator. A schematic representation of the MEW scaffold fabrication process is shown in Figure 1.

The design of each scaffold was carefully evaluated to ensure straight, uniform fiber deposition and consistent uniformly deposited fibers while maintaining a consistent porosity level across all samples. To achieve this, printing parameters, such as feeding pressure, voltage difference, print head speed, and the distance between the nozzle tip and the collector, were optimized. The optimization process was based on response surface methodology (RSM), previously published by our group,^7^ for fabricating intact 3D scaffolds with straight fibers. The interdependence of printing parameters on the fiber diameter and structural integrity was also considered. Recently, researchers have started integrating artificial intelligence (AI) into the MEW process parameter optimization. The AI models can predict the effect of the parameters on shape fidelity and fiber diameter, enabling real-time adjustments.^49,50^ These advancements hold the potential for further aligning fibers and improving deposition consistency. The specific printing parameters used in the experiments are summarized in Table 1. PCL pellets were placed in the syringe and heated for 2 h prior to printing. The heating temperature of 90 °C was maintained for all samples. Fiber spacing and structural height were set at 0.8 and 1 mm, respectively. Melt electrowritten structures were fabricated with dimensions of 20 × 20 mm and later cut into 7 × 7 mm pieces using a punch. A key challenge in melt electrowriting is stabilizing the polymer jet until it is deposited on the collector, especially when printing larger volumes. As the polymer jet contacts the collector, it disrupts the electrical conductivity between the collector and the nozzle tip. To address this, a flat glass was placed on the collector, effectively minimizing polymer jet instabilities. Additionally, dynamic parameters were carefully adjusted during the printing of honeycomb structures to ensure complete fiber alignment in each layer. To prevent unstable fiber deposition at the start and to balance the processing parameters, printing began after 2 min of polymer extrusion under high voltage. The parameters were optimized to produce structures with different geometries but consistent porosity, eliminating any potential errors caused by porosity differences.

**Table 1:** Printing Parameters for MEW of Different Geometrical Structures

scaffold geometry	printing parameters				
	printing speed (mm/min)	feeding pressure (bar)	nozzle and collector distance (mm)	acceleration voltage (kV)	number of layers
0–90° mesh	110	0.4	1.8	4	20
60–120° mesh	130	0.5	2	4	22
honeycomb	80–95	0.4–0.5	1.7–2.1	4–5	16

Fabricated scaffolds were treated with NaOH to introduce hydroxyl and carboxyl groups to the polymer, improving hydrophilicity and increasing the surface area for enhanced cell attachment.^51^ The treatment involved immersing the scaffolds in a 1N NaOH solution for 2 h at room temperature, followed by thorough washing with deionized (DI) water and PBS until the pH of the solution was neutral.

### 2.3 Cell-Laden Hydrogel Preparation

We used gelatin type B, a denatured form of bovine collagen, as a biocompatible hydrogel. Gelatin offers better mechanical and degradation properties than collagen and is nonantigenic.^52^ The cross-linking process was initiated using visible light, with Ru as the photoinitiator and SPS as the cross-linker. Gelatin, Ru, and SPS were prepared in PBS at concentrations of 10% (w/v), 20, and 10 mM, respectively, following the protocol from Advanced BioMatrix. Gelatin was first dissolved in autoclaved PBS and mixed for 1 h at 40 °C. Ru and SPS were dissolved separately in DI water and vortexed thoroughly. All solutions were then sterile-filtered using a 0.22 μm filter (Minisart).

HSFs were employed as representative skin cells, while HUVECs were used to induce capillary formation.^53^ HSFs and HUVECs were thawed at passage numbers of 10 and 7, respectively. HSFs were cultured in DMEM supplemented with 10% FBS and 1% Penn-Strep, and HUVECs were cultured in vascular cell basal medium with 10% FBS and 1% Penn-Strep. Both cell types were extracted at a concentration of 1 × 10^6^ cells/mL and mixed in a small volume of a 1:1 ratio of both media. Throughout the process, the gelatin hydrogel was kept at 37 °C. The hydrogel was prepared by adding Ru and SPS, followed by thorough mixing. Finally, the hydrogel was pipetted into the cell suspension to ensure complete mixing.

### 2.4 Hybrid Scaffold Fabrication

**Figure 2:**
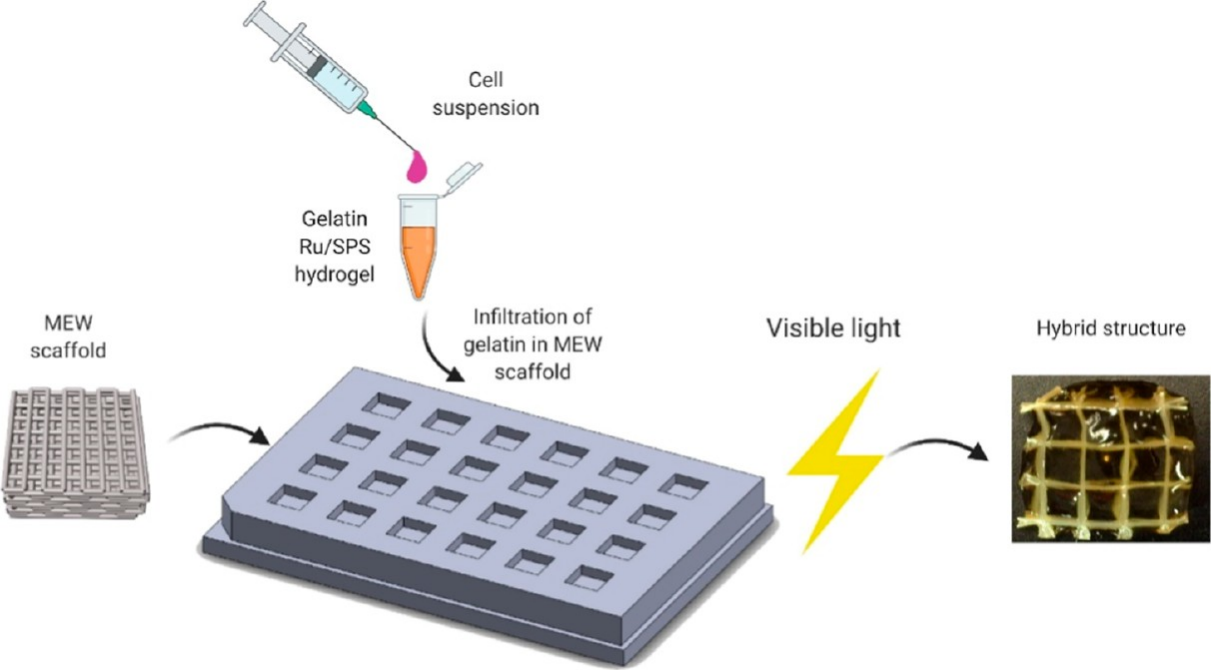
Schematic representation of the hybrid scaffold fabrication process. The melt electrowritten structure is placed within a mold, followed by the infiltration of a mixture of HSF-HUVEC within a gelatin hydrogel precursor solution containing Ru/SPS. The construct is then exposed to visible light to initiate hydrogel cross-linking.

The melt electrowritten scaffolds were sterilized by immersion in 70% ethanol for 15 min, followed by 20 min of UV exposure for each side. A sterile mold consisting of a series of 7 × 7 mm and 2 mm height cavities covered with aluminum foil was employed for hybrid scaffold fabrication. PCL structures were placed inside the molds, and cell-laden hydrogels with concentrations mentioned in the previous section were dispensed into each scaffold in equal volumes. Following casting, the hydrogels were photo-cross-linked using two 100 W white LED lamps positioned 40 cm away for 2 min. The hybrid scaffolds were then transferred to cell culture plates and incubated at 37 °C with 5% CO_2_ in a humidified environment for further analysis. The cells were cocultured in a mixed medium of DMEM and basal culture medium in a 1:1 ratio, with the medium being changed every 2 days. Figure 2 shows a schematic representation of the hybrid scaffold fabrication process.

### 2.5 Mechanical Testing

Mechanical properties of PCL and hybrid PCL-hydrogel scaffolds with various geometries were analyzed under a uniaxial tensile test using a Zwick/Roell-Z100 universal testing machine (UTM). Rectangular-shaped samples (*n* = 5), measuring 50 × 10 mm, were cut from a larger structure and tested in the longitudinal direction. A 200 N load cell with pneumatic grips was equipped for the tests, with the grip-to-grip distance set to 35 mm, and a preload of 5 kPa was applied to ensure the straightness of the structure before testing. Each sample was stretched at a rate of 2 mm/min until it reached 80% of the maximum force. The elastic moduli were calculated from the linear region of the engineering stress–strain curve obtained from the tests. Hybrid samples were analyzed immediately after cross-linking to prevent dehydration. To ensure the secure attachment of the hybrid structures to the machine grip, ultrafine sandpaper was used at the attachment points between the samples and the grips.

### 2.6 Morphological Characterization and Swelling Properties of the Scaffolds

The morphological properties of the melt electrowritten and hybrid structures were examined using a Dino-Lite digital microscope (AM4113TR4, Taiwan) and a field emission gun scanning electron microscope (FE-SEM, Zeiss Leo Supra VP 35) at an accelerating voltage of 4 kV. Prior to SEM imaging, the samples were coated with gold particles by using a Cressington 108 sputter coater.

The swelling properties of the pristine gelatin and gelatin in hybrid scaffolds were compared to assess the reinforcing effect of the melt electrowritten structures and the potential impact of the scaffold morphology on swelling. The swelling ratio was determined by immersing the scaffolds in PBS and measuring their weights before and after soaking at specific time points. The medium was changed every 3 days. The swelling ration was calculated using the following equation
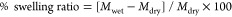
1where *M*_dry_ and *M*_wet_ are the masses of the structures before and after incubation in PBS, respectively.

The scaffolds were incubated in 1× PBS at 37 °C under continuous gentle shaking. PBS solution was prepared by dissolving tablets in Milli-Q water, and each experiment was repeated four times.

### 2.7 Cell Viability and Morphology

Viability of the cells in the hybrid scaffolds was assessed on days 1, 3, 7, and 14 using a live/dead viability assay kit, following the manufacturer’s instructions. Live cells were stained with Calcein-AM (green fluorescence, Invitrogen), while dead cells were labeled with propidium iodide (PI) (red fluorescence, Invitrogen). Briefly, the scaffolds were stained in a PBS solution containing 1 μM Calcein-AM for 30 min at 37 °C followed by a washing step with PBS and treated with a 0.75 μM PI solution in 1 × PBS for 5 min at 37 °C and finally washed with PBS three times.

To observe the morphology of the encapsulated cells after 1 week of incubation, the samples were fixed in 4% paraformaldehyde for 30 min and washed three times with PBS. Cell membranes were permeabilized with 0.1% Triton-X 100 in PBS for 10 min, followed by another wash. For F-actin staining, the samples were stained in red fluorescent Alexa-Fluor 568 phalloidin conjugate (Abcam, UK) for 1 h, protected from light, and then washed with PBS. The cell nuclei were stained with 4′,6-diamidino-2-phenylindole (DAPI) for 15 min, followed by a PBS wash. All procedures were carried out at room temperature. Tiled *z*-stacks and *z*-stacks of the structures were captured by using a Carl Zeiss LSM 710 laser confocal microscope to observe live/dead cells and cellular morphology, respectively. The percentage of viable cells was calculated using ImageJ. The images were initially processed according to the intensity. The green and red channels were separated to differentiate between the live and dead cells, respectively. The two channels were subsequently quantified individually with the Measure tool under the Analyze section. The percentage of viable cells was determined by dividing the intensity of live cells (green) by the overall intensity, which includes both live (green) and dead (red) cells. Three different confocal microscopy images were used for the analysis.

To observe the cell distribution within the hybrid structure, HUVECs (SP-DilC18, Molecular Probes) and HSFs (SP-DiOC18, Molecular Probes) were stained with red and green membrane-intercalating dyes, respectively. Prior to trypsinization, cells were stained separately with dyes (1.5 μg/mL in serum-free medium) for 30 min at 37 °C. Following staining, the cells were encapsulated in gelatin and infilled in the MEW scaffolds. The 3D images were captured after 14 days of incubation of the hybrid structures at 37 °C using a Zeiss LSM710 inverted confocal microscope.

### 2.8 Immunofluorescence Staining

The encapsulated HUVECs and HSFs within the gelatin hybrid structure were fixed with 4% (w/v) paraformaldehyde solution for 15 min at room temperature after 14 days of incubation at 37 °C. The cells were permeabilized with 0.1% (v/v) Triton X-100 in PBS for 10 min. After blocking the cells with 1% (w/v) bovine serum albumin (BSA) in PBS for one h at room temperature, the scaffolds were incubated overnight at 4 °C with CD31 antibody conjugated to Alexa Fluor 647 (Abcam, ab218582) to visualize CD31 positive HUVECs, diluted 1:100 in 1% (w/v) BSA solution. It was followed by HSFs immunostaining with CD90 antibody conjugated to Alexa Fluor 568 (Abcam, ab218582), diluted 1:100 in 1% (w/v) BSA solution for 2 h at room temperature. Cell nuclei were stained with DAPI (300 ng/mL; Invitrogen) for 15 min at room temperature. Z-stacks images were captured at 3 μm intervals using a Zeiss LSM710 inverted confocal microscope.

#### 2.8.1 Statistical Analysis

For the cell study tests, 4 replicates were used. The mechanical tensile test was performed with 5 replicates. Student’s *t*-test was carried out in GraphPad Prism 5. Differences were considered significant with a p-value smaller than 0.05. ^★^ indicates *P* < 0.05, ^★★^*P* < 0.01, and ^★★★^*P* < 0.001. Data are represented as mean ± standard deviation.

## 3 Results and Discussion

### 3.1 MEW of Scaffolds with Various Geometries

**Figure 3:**
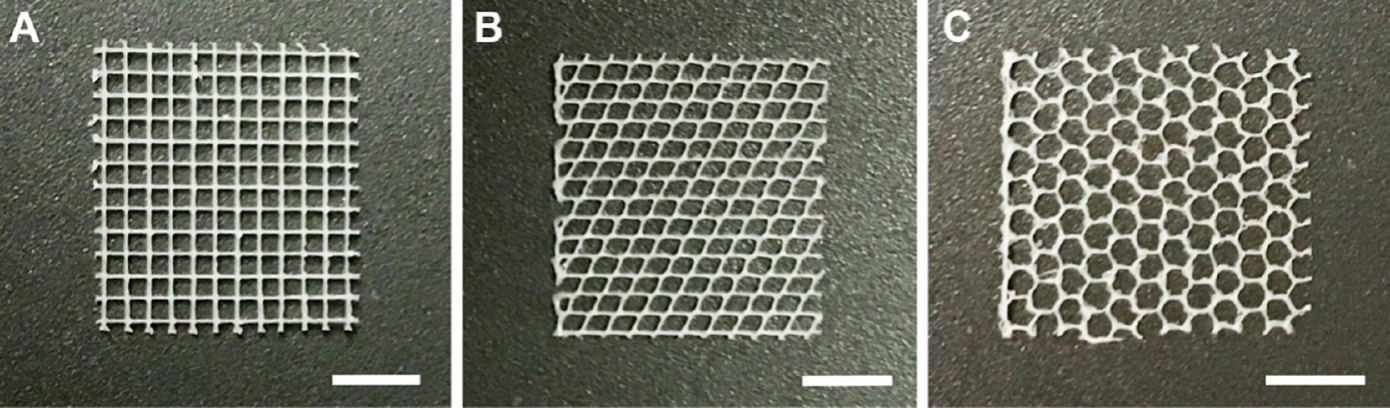
Digital microscope images of melt electrowritten scaffolds. (A) 0–90° mesh, (B) 60–120° mesh, and (C) honeycomb structures. Scale bars are 5 mm.

MEW is a benchmark for scaffold fabrication that enables the reduction of fiber diameter to a few or tens of microns with orderly controlled filament deposition.^54^ Despite the progress made, technical challenges still remain to be addressed and developed for long processes with complex architectures and high-layer numbers. To adaptively control the polymer jet during printing, dynamic parameter optimization could be utilized as explained by Jin et al.^54^ Here we created tool path planning for three different structures with various geometries aiming to investigate the effect of fiber direction in cellular alignment and morphology, which could result in lumen structure formation for vascularization as a critical factor of volumetric structural design. The morphologies of the MEW scaffolds were examined using a digital microscope as shown in Figure 3.

### 3.2 Mechanical Analysis of the Scaffolds

**Figure 4:**
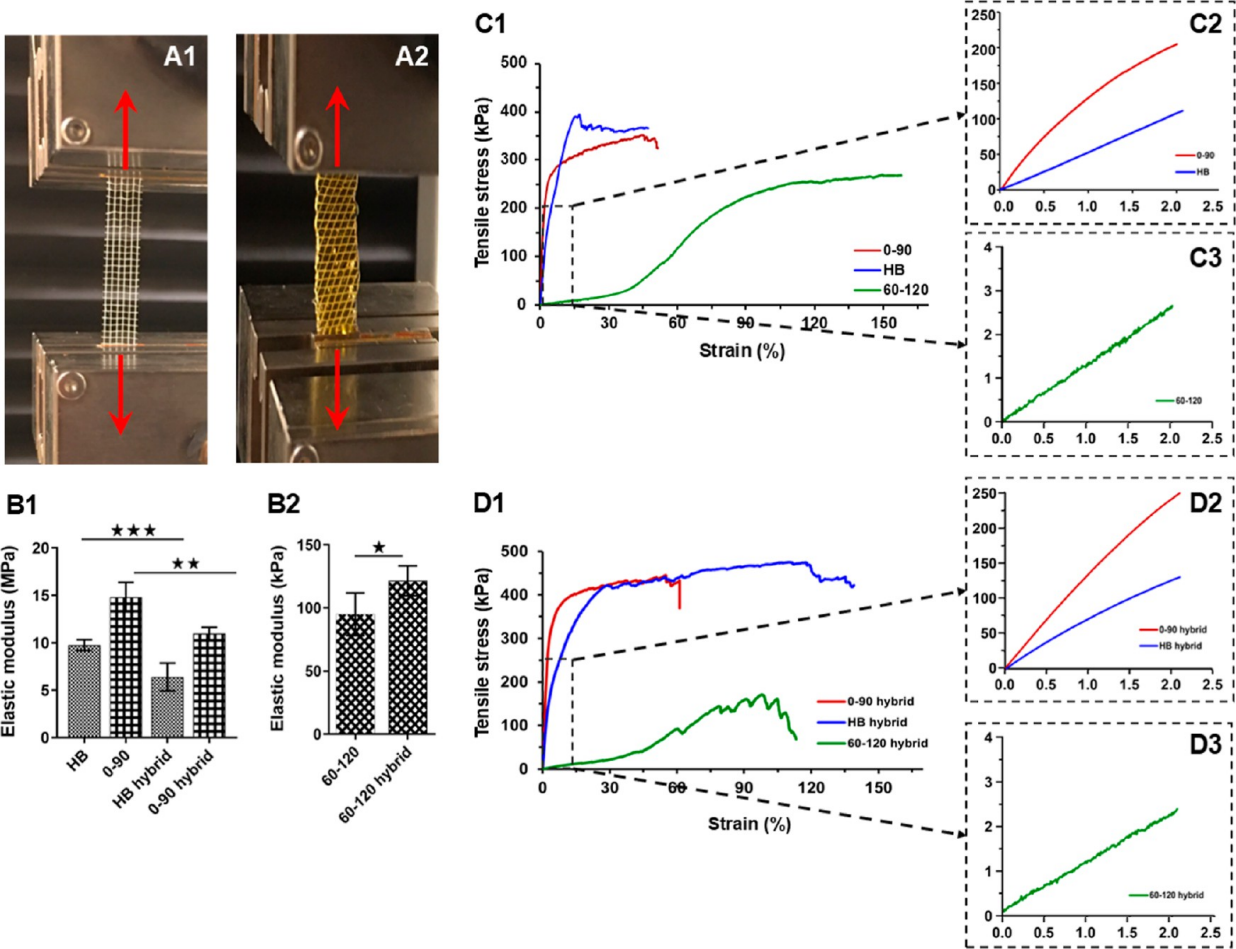
Mechanical properties of the MEW fabricated PCL and PCL-hydrogel hybrid scaffolds. Representation of the experimental setup for the (A(1)) 0–90° scaffold and (A(2)) hybrid 60–120° scaffold under uniaxial strain. (B(1,2)) show elastic modulus values of all the structures calculated from the linear region of the stress–strain curves. Representative stress–strain curves of scaffolds with different geometries for (C) PCL and (D) PCL-hydrogel. Insets showing strain values up to 2%. ^★^ indicates *P* < 0.05, ^★★^*P* < 0.01, and ^★★★^*P* < 0.001.

The mechanical properties of the melt electrowritten scaffolds were characterized by measuring the elastic modulus and elongation at break using a uniaxial tensile test. MEW and hybrid structures were examined separately to investigate the effects of geometry and the presence of the hydrogel. Elastic modulus and elastic energy are critical for skin tissue substitutes, as these materials will be subjected to axial tensile forces. It is important to note that these properties may vary based on factors such as age, sex, and the specific location of skin in the body for which the scaffolds are intended. As previous studies have suggested,^12,55^ fibrous structures can enhance the mechanical properties of soft network hydrogels. Therefore, the hydrogel samples were not characterized separately as a control group. Different geometries were investigated, both with and without hydrogel, and their elastic moduli (*E*), elastic energy (*U*_elastic_), and elongation at failure were measured and compared. Figure 4 illustrates the stress–strain curves and the corresponding analyses of the elastic moduli. Stiffness values were obtained by calculating the slope of the linear region of stress–strain curves. The honeycomb and 0–90° mesh structures showed similar mechanical properties when compared to those with a 0–60° mesh. Table 2 presents the elastic moduli, elastic energy calculated from the area under the stress–strain curve, and elongation at failure (ε_f_) for all of the tested geometries.

**Table 2:** Mechanical Properties of the Scaffolds Obtained from the Tensile Test

	structures					
	only PCL			PCL/hydrogel		
	0–90°	60–120°	honeycomb	0–90°	60–120°	honeycomb
E (MPa)	14.54 ± 1.58	0.095 ± 1.31	9.76 ± 0.51	10.95 ± 155	0.12 ± 1.17	6.40 ± 1.32
*U*_elastic_ (kJ/m^3^)	106.10 ± 4.0	7.43 ± 2.1	174.71 ± 3.4	82.33 ± 4.7	37.13 ± 3.6	121.37 ± 5.2
ε_f_ (%)	51.5 ± 2.8	242 ± 4.4	46.48 ± 2.6	61.2 ± 3.8	113.1 ± 2.4	138.2 ± 4.5

Figure 4A(1,2) presents representative images of MEW PCL with a 0–90° mesh and a hybrid structure with a 60–120° mesh under uniaxial strain, respectively. A comparison of the Young’s moduli of samples with different internal architectures indicates that the 0–90° mesh structure exhibited the highest elastic modulus (Figure 4B(1)). However, it failed with a minimum plastic strain. This behavior is likely due to the fibers being fully aligned with the direction of the applied load, while the perpendicular joints restricted further movement and plastic deformation of the structure. A similar pattern was observed for the samples with a honeycomb geometry. In contrast, the 60–120° mesh demonstrated lower stiffness compared to both structures [Figure 4B(2)]. This is attributed to the inclined orientation of their fibers relative to the applying force. This inclined configuration provides enhanced stretchability compared to the 0–90° mesh.^56^ The 0–90° mesh displayed a rapid increase in stress, as its tightly packed fibers were less flexible in the lateral direction [Figure 4C(1,2)].^57^ Furthermore, the 0–90° mesh entered the plastic region at a much slower strain value compared with the honeycomb structure. This can be explained by its structural ability to withstand more elastic deformation both in the direction of elongation and perpendicular to it.^56,58^ In contrast, the samples with 60–120° mesh structures exhibited a significantly lower elastic modulus while withstanding a large plastic strain of up to 120% [Figure 4C(1,3)]. The presence of fibers oriented at 60 and 120° relative to the applied force led to a gradual rearrangement of the structure during the initial loading phase. Additionally, the stretching of fiber junction points in the direction of the applied force enabled greater plastic deformation of the structures without causing significant failure in the load-bearing fibers. Furthermore, the 60–120° mesh yielded higher strain values compared to both the 0–90° mesh and honeycomb structure.

The hybrid structure exhibited a greater plastic deformation effect on both the 0–90° mesh and honeycomb structures [Figure 4D(1)]. This can be attributed to the incorporation of hyperplastic hydrogel within the matrix, which facilitated force distribution from the stiff fibers to the soft and flexible hydrogel, leading to greater deformation upon failure.^57^ In contrast, the hybrid samples with a 60–120° fiber orientation experienced an elevation in the elastic modulus [Figure 4D(2)]. This may be due to the fact that only the PCL structure exhibited an elastic modulus comparable to that of the hydrogel itself. Consequently, the incorporation of the hydrogel into the fibrous structure resulted in load transfer to both the soft and hard matrices, ultimately increasing the elastic modulus.^43^ Moreover, failure in the plastic region occurred at lower strain values for hybrid samples with 60–120° structures. This could be attributed to the lower lateral expansion capacity of the hydrogel compared to the fibrous mesh, causing the hybrid structure to rupture at lower displacements.^59^ The energy of the elastic region was also calculated from the integration of the elastic region of the stress–strain curve from zero strain until it passed the linearity. As shown in Table 2, the elastic energy of the 0–90° mesh and honeycomb samples decreased in their hybrid forms, following the same trend as the elastic modulus values. However, the energy stored in the 60–120° structure in the elastic region increased significantly (almost 5-fold).

Indeed, the final mechanical properties of such complex samples are strongly influenced by structural characteristics, such as fiber diameter, fiber spacing, porosity, fiber orientations, structural side length, and the number of layers. Therefore, results may vary compared to other studies.^60,61^ This variety of properties offers the potential for designing structures tailored to specific applications and tissue properties. It is worth noting that skin is a heterogeneous tissue with a wide range of mechanical properties, including elastic modulus and elongation at failure.^62^ Thus, these varying results could be beneficial for skin applications in different areas of the body. The results in Table 2 demonstrate that elastic modulus values for both the 0–90° and honeycomb structures fall within the range of 4–20 MPa, which is consistent with values typically reported for the dermis layer of human skin in tensile testing.^63^

### 3.3 Morphological Characterization and Swelling Properties of the Scaffolds

**Figure 5:**
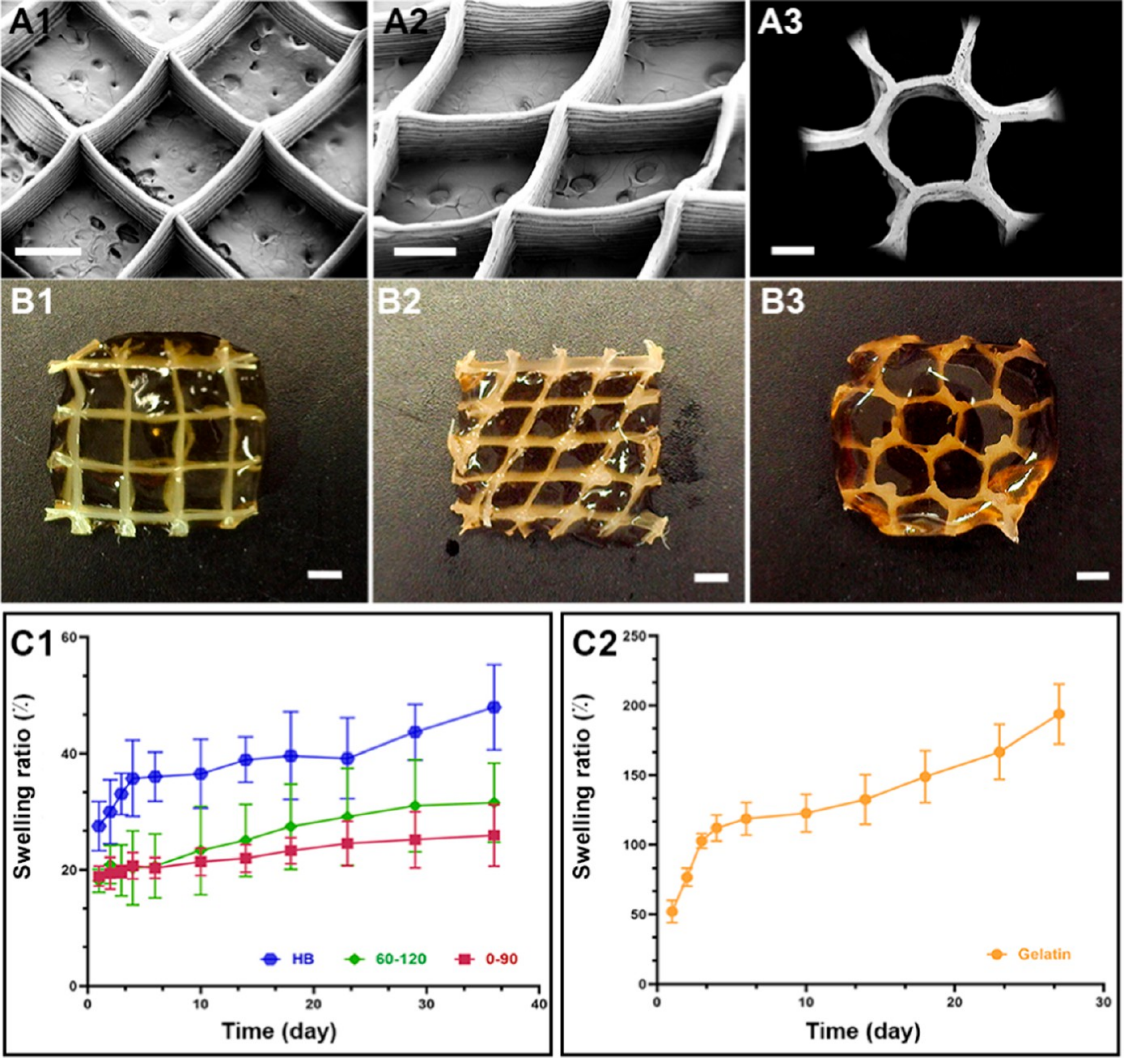
Morphological characterization of melt electrowritten scaffolds and swelling properties of the gelatin in pristine and hybrid scaffolds (A) SEM images of the melt electrowritten scaffolds. (B) Hybrid structures fabricated by infiltration of gelatin hydrogels within the fibrous scaffolds. (C) Swelling ratio of (C1) hybrid scaffolds and (C2) pristine gelatin hydrogel in PBS at different incubation times. Scale bars: (A) 0.5, and (B) 1 mm.

The morphologies of the MEW scaffolds were examined using SEM to assess structural integrity and measure fiber diameter (Figure 5A). Fiber diameters were calculated using ImageJ software by analyzing 40 randomly selected fibers at various points, both near and far from joint points. The average fiber diameters for the 0–90° mesh, 60–120° mesh, and honeycomb structures were 79.81 ± 3.9, 80.20 ± 6.53, and 116.11 ± 9.10 μm, respectively. The honeycomb structure exhibited a higher standard deviation value due to its geometric complexity, with multiple short turns causing greater variation in filament diameter compared to the mesh structures (Figure 5A). Cell-laden hybrid structures were also visualized by using a Dino-Lite digital microscope (Figure 5B).

A swelling test was conducted on both hybrid and pristine gelatin samples. The swelling ratio of pristine gelatin hydrogel rapidly increased within the first 3 days (Figure 5C). In contrast, the hybrid scaffolds demonstrated a significant lowering in swelling properties of gelatin, even after 35 days of incubation in PBS. While mesh structures displayed similar swelling behavior, the hybrid scaffold with honeycomb geometry showed greater swelling due to the large pores between PCL filaments, which were filled with the hydrogel. These results underscore the substantial impact of the PCL filament geometry on the stability of the hydrogel in hybrid structures.

### 3.4 Cell Viability and Morphological Analysis

**Figure 6:**
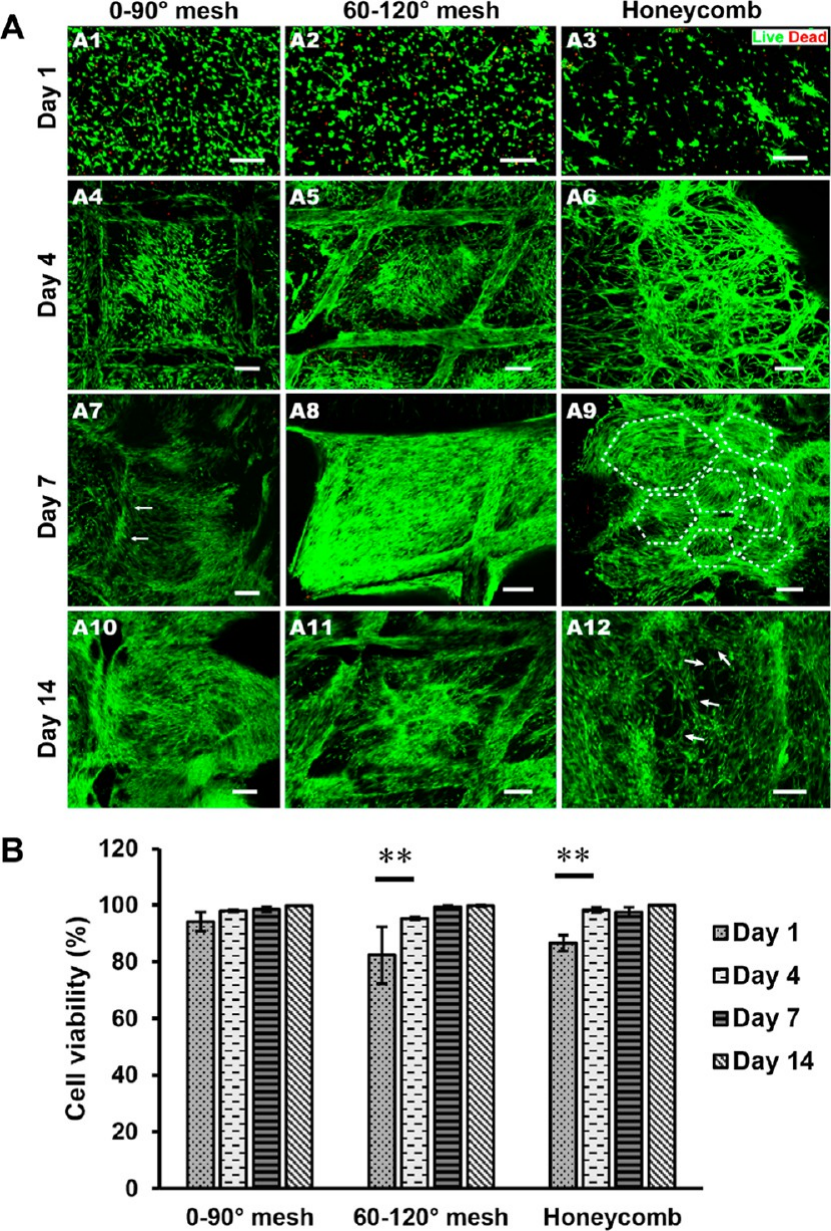
(A) Confocal microscopy images of HSFs and HUVECs encapsulated in gelatin hydrogel in the 0–90° mesh, 60–120° mesh, and honeycombs hybrid scaffolds. (calcein-AM (green) and PI (red) staining performed at (A1–3) day1, (A4–6) day 4, (A7–9) day 7, and (A10–12) day 14 of incubation to show live and dead cells. Scale bars are 200 μm. (B) Quantitative analysis of cell viability. Cell viability was assessed at various points by measuring the intensity of green/red fluorescence using ImageJ software. The percent cell viability was calculated as the ratio of green fluorescence intensity to the total fluorescence intensity (green + red), multiplied by 100. The analysis was performed on three independent confocal microscopy images for each time point. Statistical differences were analyzed using Tukey’s multiple comparisons test with two-way ANOVA (error bars: ±SD, **P < 0.01).

The properties of the 3D extracellular matrix, particularly its physical structure and biochemical composition, are crucial regulators of cell–matrix and cell–cell communication. These interactions play an important role in the construction of skin substitutes and development of vascularized skin substitutes. In this study, HUVECs and HSFs were utilized as representative endothelial cells for vascularization and dermis abundant cells, respectively. To investigate the cytocompatibility and the effects of geometrical cues on cellular behavior, melt electrowritten structures in 0–90 and 60–120° mesh patterns were infiltrated, with HUVECs and HSFs encapsulated in gelatin hydrogel. Over a period of 2 weeks, the cultured structures were examined at specific time points (days 1, 4, 7, and 14) using the Calcein-AM/PI assay for live/dead analysis. Figure 6 presents confocal images of the scaffolds stained with Calcein-AM/PI.

On the first day of incubation, confocal microscopy images revealed that the viable cells were homogeneously distributed throughout the scaffolds (Figure 6A). Cell viability for the 0–90° mesh, 60–120° mesh, and honeycomb structures was calculated as 94.2 ± 3.4%, 82.4 ± 10.0%, and 86.6 ± 2.83%, respectively (Figure 6B). Over time, the cells began to spread and formed a spindle-like morphology within the gelatin hydrogel-infiltrated melt electrowritten structures across all geometries. These results indicate that the engineered hybrid structure provides a cytocompatible environment where the cells can maintain viability of over 90% over 14 days of incubation time.^64^ After 4 days of incubation, the cells aligned themselves along with the ordered architecture of the scaffolds [Figure 6A(4–12)], demonstrating the influence of scaffold morphology on promoting cell alignment and organization.

The distinct cell alignment appeared to be driven by geometric factors as the incubation time progressed. Most cells preferred to attach to the PCL filaments, while others in the gelatin hydrogel aligned toward the PCL filaments. As a result, cells are oriented differently depending on the scaffold geometry. In the 0–90 and 60–120° mesh structures, cell alignment was parallel to the fiber, whereas in the honeycomb scaffolds, cells aligned in a rounded pattern along the PCL fibers. Interestingly, this cell alignment led to the formation of small polygonal-like shapes within the hydrogel portion of the honeycomb structure, highlighted with white dashed lines in Figure 6A(9). These small polygons, with dense cell populations at the edges, may facilitate subsequent formation of cord-like structures.^36^ After 2 weeks of incubation, the cells in the honeycomb developed vessel-like structures primarily within the hydrogel matrix (indicated by white arrows in Figure 6A(12), with some empty spaces potentially providing room for vessel development. Notably, previous studies have demonstrated that coculturing of fibroblasts with HUVECs enhances the formation of cord-like structures.^64,65^

**Figure 7:**
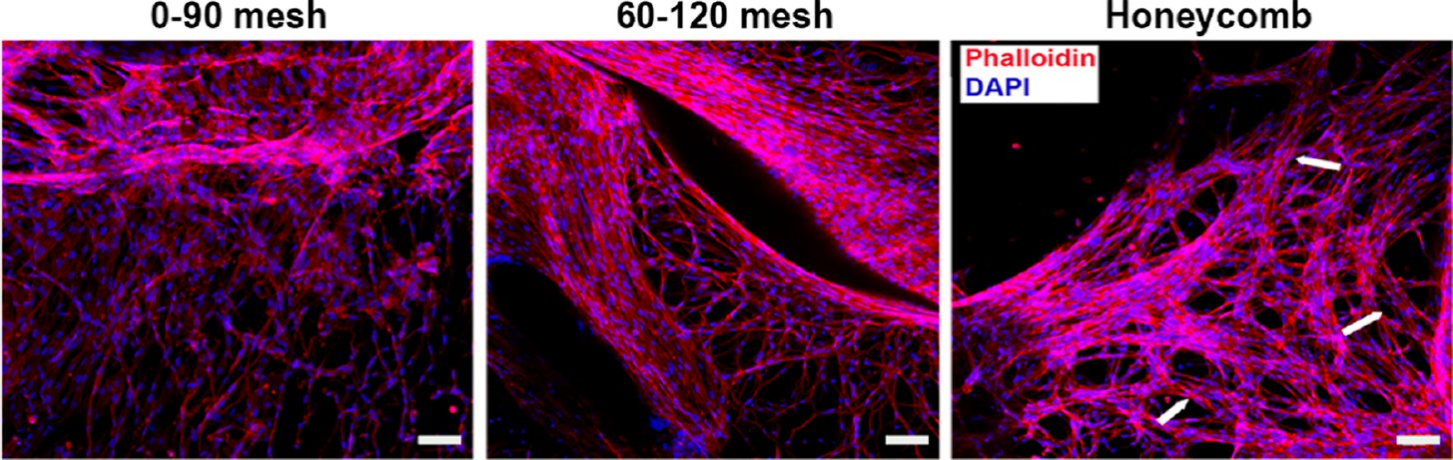
Fluorescence images of 0–90° mesh, 60–120° mesh, and honeycomb structures stained for F-actin (phalloidin: red) and nuclei (DAPI: blue) on day 7 of incubation. Scale bars are 100 μm.

On day 7, the cytoskeleton and nuclei of the cell were stained with phalloidin (red) and DAPI (blue) for detailed morphological analysis to assess the geometrical influence of the scaffolds. As shown in Figure 7, spindle-shapes cells on the 0–90° mesh structure were predominantly spread across the scaffold without a clear preferential orientation. In contrast, the 60–120° mesh exhibited more cells attached to the PCL walls with the cells within the hydrogel aligning according to the fiber angles. For the honeycomb structure, cells exhibited a more selective spreading pattern, forming a tubular morphology that could potentially contribute to vessel formation. Cord-like structures within the honeycomb scaffold are highlighted by white arrows in Figure 7. These findings demonstrated that cells adapt to the microenvironment with a proper morphology, and migrate and align.^41,66^ Our results suggest that the honeycomb structure has potential as a scaffold to facilitate vascularization, particularly in thick skin substitutes.

In this study, HUVECs were selected as they are widely regarded as a standard model for evaluating vascularization and angiogenesis in 3D systems due to their numerous advantages.^67−69^ They are relatively easy to isolate, cost-effective, exhibit robust angiogenesis capabilities, express key endothelial markers, and are responsive to proangiogenic factors forming capillary-like structures in vitro. Co-culturing them with other cell types such as fibroblasts or mesenchymal stem cells makes them ideal for standardized vascularization assays. However, they also have notable limitations. As primary cells, HUVECs show short lifespan and limited proliferation capacity. Their venous origin restricts their ability to generalize a conclusion for other vascular regions such as arteries and capillaries. Additionally, while HUVECs form tube-like structures in 3D culture, the networks could lack maturity and stability as seen in vivo.^67,69,70^

**Figure 8:**
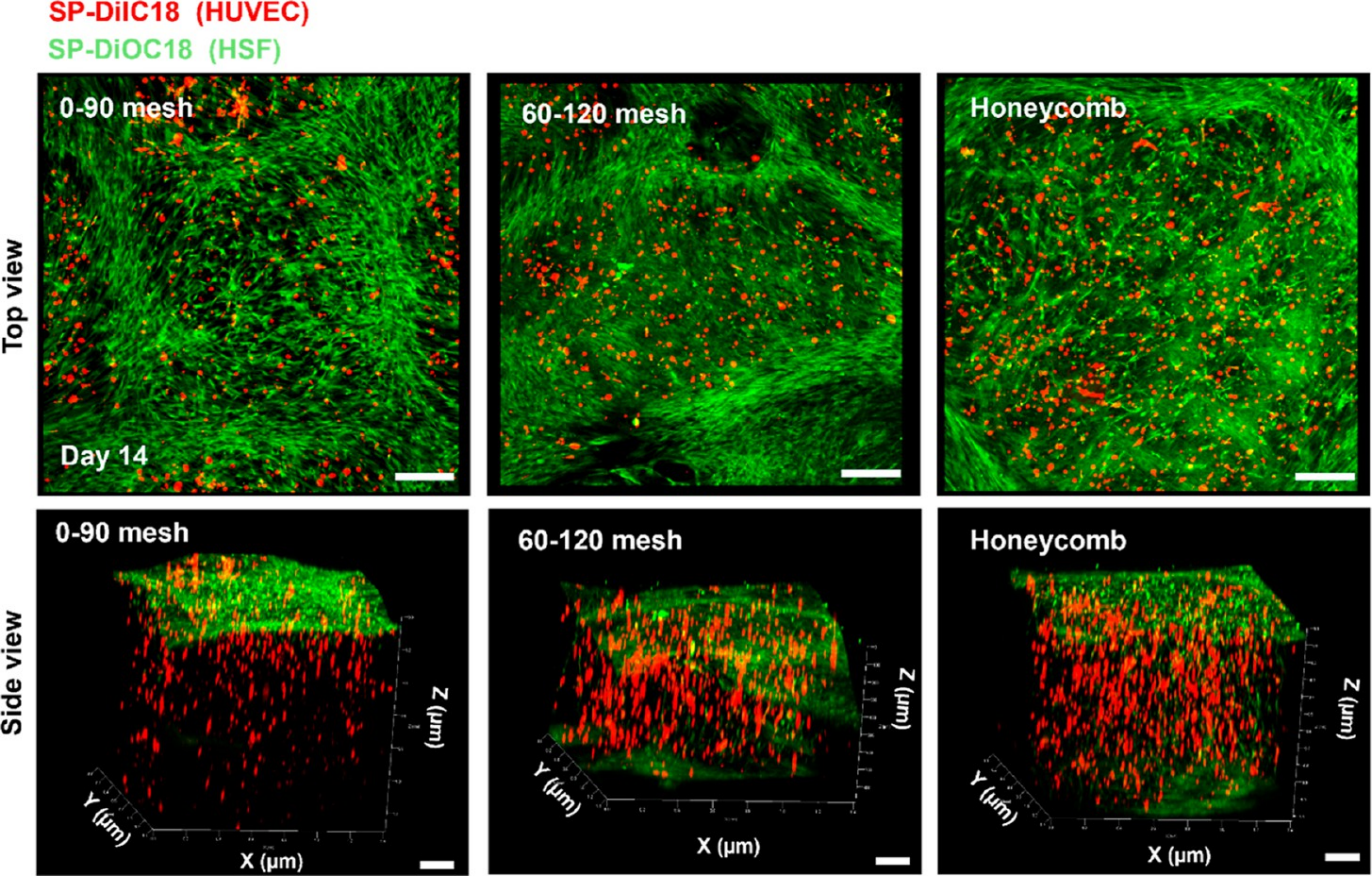
Confocal microscopy images of HUVECs and HSFs infilled in 0–90° mesh, 60–120° mesh, and honeycomb MEW scaffolds. HUVECs and HSFs are labeled with SP-DilC18 (red) and SP-DiOC18 (green) in the scaffolds and imaged on the 14 day incubation period. Z-stacked images are shown from top and side views. Scale bars represent 200 μm.

Cells exhibit complex and dynamic functions within a 3D matrix, influenced not only by their intrinsic characteristics but also by the physical properties of their local environment.^71−75^ Additionally, interactions with neighboring cells and the ECM significantly affect behaviors, such as migration, proliferation, and tissue remodeling during regeneration. However, a lack of a comprehensive understanding of these multifaceted cell–cell and cell-ECM interactions, as well as their dynamic interplay within a 3D matrix, remains a considerable challenge in fully reconstructing skin function. We investigated the distribution and orientation of HSFs and HUVECs within various 3D hybrid structures after a 14 day incubation period. Uneven distribution of HSFs and HUVECs in the hybrid scaffolds was apparent across all MEW geometries as shown in Figure 8. This behavior is likely due to the differing migratory tendencies of the two cell types.^76,77^ Fibroblasts typically exhibit mechanosensitive behavior by preferentially migrating toward stiffer regions of the ECM in response to their microenvironment.^75,78^ In our hybrid scaffolds, this mechanosensitive response was evident, as HSFs (green) predominantly localized to the periphery surface of the hybrid scaffold, primarily on the stiffer PCL MEW fibers. This localization could be advantageous for keratinocyte migration and epithelization in later stages of skin regeneration, as stiffer surfaces activate fibroblast migration and promote higher expression of fibrous ECM proteins.^75,78^ Furthermore, the angular geometry at the cross sections of the MEW fibers provided directional guidance for HSF elongation toward the gelatin hydrogel.

**Figure 9:**
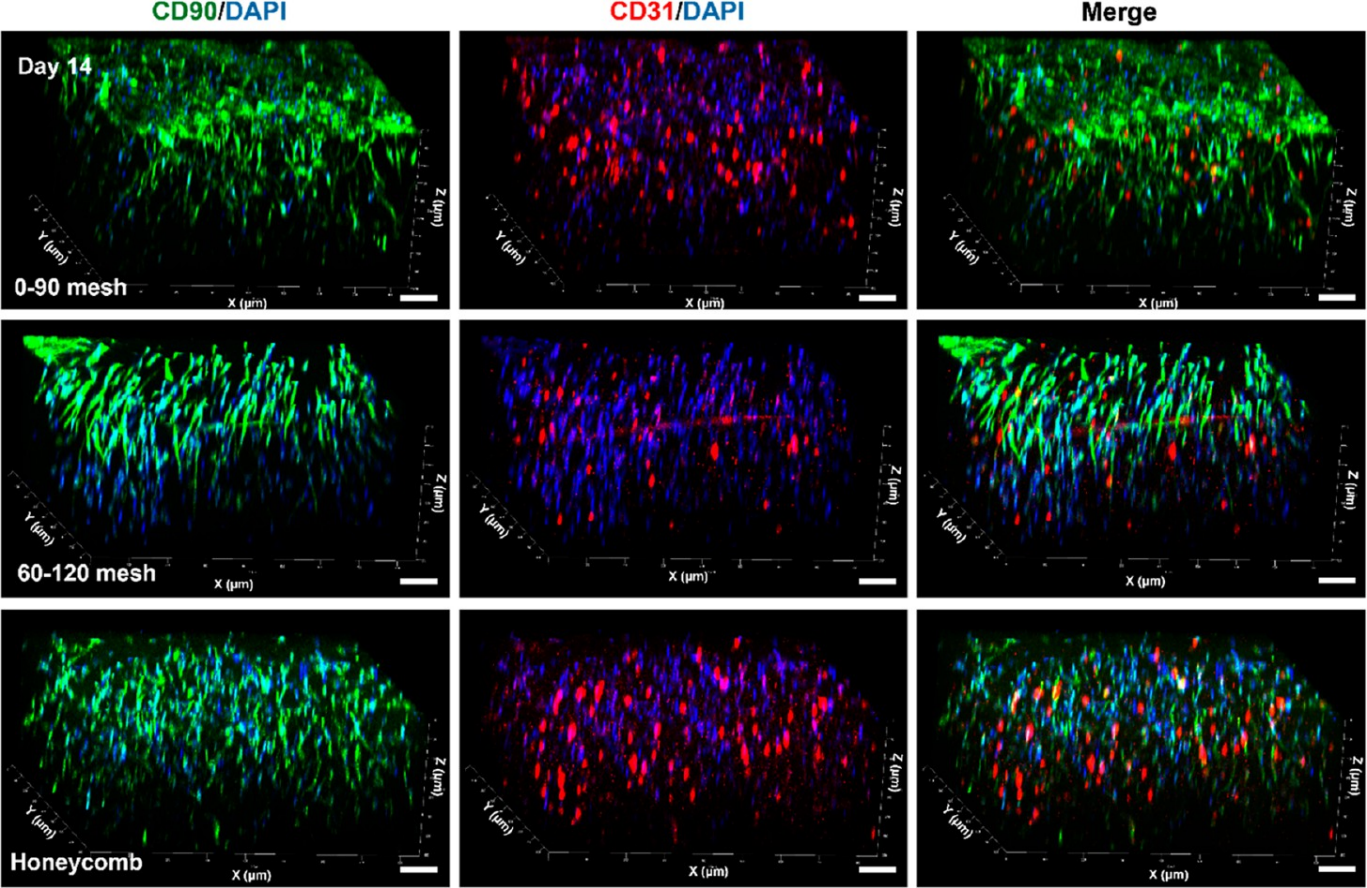
Immunofluorescence staining images of HUVECs and HSFs embedded within the hydrogels in 0–90° mesh, 60–120°, and Honeycomb scaffolds after 14 days of incubation. CD31 positive HUVECs are visualized in red, and HSFs are represented with CD90 positive cells in green. Cell nuclei are counterstained with DAPI (blue). Scale bars are 100 μm.

HUVECs (red) were predominantly distributed throughout the gelatin matrix infiltrated within MEW PCL fibers. Geometrical guidance, along with HSF-matrix and cell–cell interactions, likely contributed to the formation of complex networks and influenced the downstream tubular morphogenesis of HUVECs.^79^ CD90-positive fibroblasts are essential in stimulating in situ angiogenesis by regulating physiological processes and capillary morphogenesis.^80^ CD31, a protein that is highly expressed by endothelial cells at cell–cell contact areas, serves as a key marker for capillary formation. To evaluate the influence of scaffold geometry on capillary morphogenesis in thick skin substitutes, we analyzed the expression profiles of CD90 and CD31 in HSFs and HUVECs, respectively. The elongation behavior of CD90 positive (green) HSF across different scaffold geometries influenced downstream morphological changes and the subsequent expression of CD31 (red) in HUVECs, indicating an angiogenic activity. Fibroblasts play a critical role in regulating tube assembly, remodeling, and stabilization of HUVECs by secreting growth factors such as fibroblast growth factor (FGF) and vascular endothelial growth factor (VEGF).^79,80^ Notably, CD31 expression in HUVECs was evident after a 14 day incubation period (Figure 9). The growth factors secreted by HSFs are expected to be thoroughly distributed within the hydrogel, promoting neovascularization and enhancing the potential of the hybrid scaffolds for thick skin tissue regeneration.

## 4 Conclusions

In this study, we demonstrated the effect of geometry on the mechanical properties of the scaffolds and cellular behavior in three distinct MEW PCL mesh geometries: 0–90° mesh, 60–120° mesh, and honeycomb structures within a hybrid scaffold, highlighting their potential use in thick skin substitutes. Among the three PCL structures, the honeycomb geometry exhibited a high elastic modulus, elastic energy, and strain values upon failure, which are compatible with skin tissue. Regardless of the geometry, all three structures supported a high cell viability and homogeneous cell distribution within the hybrid scaffolds. Cellular alignment results indicated that the 0–90° mesh and honeycomb structures were particularly effective in guiding cocultured cells, with the greatest influence on upregulation of CD31 expression, as a marker of vascularization, compared to the 60–120° mesh. Overall, the developed honeycomb MEW-based hybrid structure demonstrated effective promotion of vascularization and shows potential for improving the healing of injured skin tissue. We anticipate that the functionality of this hybrid system for full-thickness skin tissue engineering could be further enhanced by strategically modifying both the MEW PCL scaffold and hydrogel components with additional bioactive molecules. These future optimizations could expand the therapeutic potential of this hybrid approach for skin tissue applications.
